# Surface‐guided analysis of breast shape changes during postoperative radiotherapy by using a functional map framework

**DOI:** 10.1002/mp.70532

**Published:** 2026-06-30

**Authors:** Pierre Galmiche, Hyewon Seo, Yvan Pin, Philippe Meyer, Georges Noël, Michel De Mathelin

**Affiliations:** ^1^ Laboiratoire ICube CNRS–University of Strasbourg Strasbourg France; ^2^ Institut Privé de Radiothérapie de Metz (IPRM) Metz France; ^3^ Institut de Cancérologie de Strasbourg Europe (ICANS) Strasbourg France

**Keywords:** breast shape changes, post‐operative radiotherapy, shape analysis

## Abstract

**Background:**

While positioning errors during radiotherapy are increasingly well‐characterized and accounted for in the Planning Target Volume (PTV), little is known about the shape and volume changes of the breast in the course of radiotherapy. Yet they represent an unknown factor that may impact treatment effectiveness, highlighting the need to analyze inter‐fractional breast deformations to enable promising approaches such as patient‐specific adaptive irradiation planning.

**Purpose:**

In this study, we develop a geometric approach to analyze inter‐fractional breast deformation throughout the radiotherapy treatment, based on a dataset of 3D surface scans of patients' frontal torso acquired during radiotherapy sessions.

**Methods:**

We used a handheld 3D range scanner to collect surface scans of 22 patients prior to, during, and after radiotherapy sessions following breast‐conserving surgery (lumpectomy). We begin by adapting functional map framework to compute inter‐ and intra‐patient nonrigid correspondences. To study shape changes during radiotherapy, we then perform both intrinsic and extrinsic analyses of the collected breast surface shapes, by leveraging the intra‐ and inter‐patient correspondences. These analysis provide complementary insights into how the breast changes over time: The intrinsic analysis evaluates deformation patterns directly on the shape manifolds, enabling the identification of regions exhibiting high conformal (bending) or area distortions (stretching). We also perform extrinsic analysis, where we align surface acquisitions of the treated breast with the CT‐derived skin surface to assess pointwise surface displacements and volume changes in the treated area, thereby producing quantitative measures such as displacement amplitudes (in mm) and relative volume changes (in percentage).

**Results:**

The qualitative shape collection analysis highlight deformations in the contra‐lateral breast and armpit areas, along with positioning shifts on the head or abdominal regions. On average, displacements within the treated breast exhibit amplitudes of 1–2 mm across sessions, with higher values observed at the time of the 25th irradiation session. Volume changes, inferred from surface variations, reached up to 10% over the course of treatment, with the majority of values ranging between 2% and 5%.

**Conclusions:**

A ‐based geometric approach has been proposed for analyzing breast shape changes over the course of radiotherapy from surface data acquisitions. Based on a functional map framework, we develop a shape matching specialized for the breast data as well as a shape variability analysis within a unified space. Analysis across multiple patient datasets revealed a wide spectrum of breast changes despite a clinically acceptable quantitative average, demonstrating the power of our method to perform both quantitative and qualitative analysis of deformation patterns both on the individual and group level.

**Trial Registration:**

The clinical trial data used in this paper is registered under the ClinicalTrials.gov ID NCT03801850.

## 1 INTRODUCTION

Regular clinical examinations of patients during post‐operative breast radiotherapy show frequent cases of progressive changes in breast shape. T. Alderliesten et al.^1^ estimated inter‐fractional breast variations in the order of 4 mm. Seppälä et al.^2^ observed a maximum breast surface expansion up to 15 mm with 17% greater than 5 mm, suggesting the need for an additional 8 mm of margin to cover the whole breast in 95% of the treated fractions in their study. These changes may be attributed to seroma involution,^3^ or oedema/inflammation phenomena.^4^, ^5^, ^6^ While the exact mechanisms behind the shape changes remain uncertain, a systematic analysis of alterations in breast shape and volume is essential for assessing their subsequent impact on treatment quality.

Surface data is increasingly used within the treatment of breast cancer, with clinical utility demonstrated for initial patient positioning, real‐time motion monitoring, and beam gating in a variety of anatomical sites.^7^ A notable advancement is the recent surface‐guided radiotherapy (SGRT), which leverages frequently measured surface data during treatment to prevent irradiation when patient positioning errors are unacceptable. Despite its advantages in terms of accuracy, and speed,^8^, ^9^, ^10^ it remains relatively costly and not widely accessible, preventing the medical community from fully exploiting the advances in 3D geometric processing. More importantly, little research has been devoted to measuring and analyzing the shape changes in breast shape throughout the course of radiotherapy.

In this paper, we introduce the first comprehensive study based on the geometric analysis of a surface dataset to characterize shape variability across treatment sessions and patients following breast‐conserving surgery. In a previous study, Gorecki et al.^11^ proposed an approach to modify the CT volume so that it takes into account the SGRT surface deformations. However, their method focuses primarily on individual cases. In contrast, our work addresses this gap by introducing the first methodology that uses functional maps to perform such an analysis on collections of breast radiotherapy surface data.

## 2 DATA AND METHODS

### 2.1 Dataset

Our patient's surface data collection protocol has been approved by the Ethics Committee for the Protection of Individuals at the Faculty of medicine of the University of Strasbourg, which involved the written informed consent from all patients in this study (No RCB: 2017‐A02489‐44). To systematically study breast shape changes throughout the radiotherapy process and during the post‐treatment period, surface scans of patients undergoing post‐operative radiotherapy were acquired as part of a clinical trial conducted by the Institute of Cancerology Strasbourg Europe (ICANS), registered under the ClinicalTrials.gov ID NCT03801850.

**FIGURE 1:**
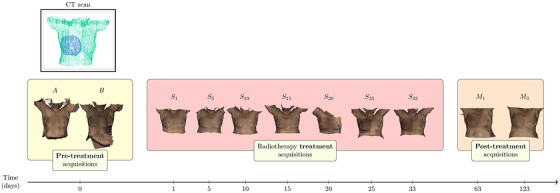
Illustration of our clinical trial data acquired for each patient.

Each patient underwent a standard course of 25 sessions of conventionally fractionated breast radiotherapy with 2 Gy per session, followed by a boost dose delivered in 8 additional sessions of 2 Gy each. During each scan, the operator swept the hand‐held scanner slowly over the torso while paying attention to the coverage of the breasts. An Artec Eva structured‐light 3D scanner was used, which operates at up to 16 fps and offers a spatial resolution of 0.1 mm with a 3D point accuracy of approximately 0.2 mm. Although its frame rate is lower than that of typical SGRT systems (35 fps), it provides comparable precision (0.1 mm) at substantially lower cost. As illustrated in Figure 1, surface scans are categorized as pre‐treatment, treatment, or post‐treatment based on the time of acquisition. (1) Pre‐treatment: To assess measurement repeatability, two scans were acquired immediately after the planning CT. (2) Treatment: One scan was performed every five sessions, resulting in 5 to 7 scans per patient. (3) Post‐treatment: Two additional scans were obtained one and three months after the end of radiotherapy to monitor longer‐term changes. In total, 247 surface acquisitions were available for analysis, reflecting the 8 to 11 scans obtained per patient across the 22 patients. Each patient's data consists of two types:

- **The CT skin surface** is reconstructed as a surface mesh from a point cloud (Figure 1, top row) derived from the skin surface in *planning CT scan* image, where the target breast volume and organs at risk (OAR) are annotated by clinicians.
- **Surface scans** are obtained as triangular meshes with about 10,000 vertices (Figure 1, bottom row), following the simplification of the initial mesh captured with the hand‐held scanner. These meshes represent different coverages of the patients' frontal torso, are not aligned, and contain noise.

### 2.2 Overview

**FIGURE 2:**
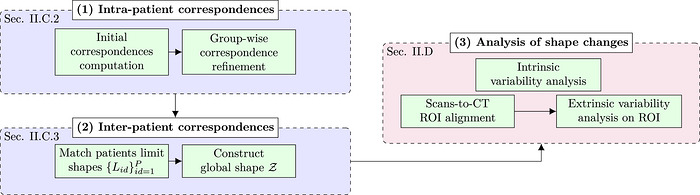
Schematic illustration of our approach and its components.

We developed a complete workflow for analyzing and modeling changes in patients' breast shape during radiotherapy from the surface acquisitions described in Section 2.1. Establishing correspondences between these surfaces is essential for accurate comparison. To achieve this, we leverage the powerful Functional Maps framework,^12^ which operates in the spectral domain by representing functions on surfaces using a basis of eigenfunctions of the Laplace‐Beltrami operator. These eigenfunctions form a natural, ordered basis that is intrinsic and invariant to pose, making them particularly well‐suited for analyzing nonrigid shape variations. By shifting the problem from establishing point‐to‐point correspondences to finding a transformation between function spaces, the framework enables the matching to be formulated as a compact linear map that preserves structural relationships between the shapes. More details on Functional Maps are provided in Appendix A.1. The proposed workflow consists of two main steps:

- **Inter‐collection shape matching**: We first compute *intra‐patient* correspondences among surface acquisitions from individual patients (Figure 2(a)), which are then used to establish *inter‐patient* correspondences (Figure 2(b)).
- **Inter‐collection shape analysis**: We then perform inter‐collection shape analysis by examining shape variability with a focus on the breast region (Figure 2(c)):
  – Intrinsic (qualitative) variability: By leveraging the functional map representation of breast shapes from the previous step, we exploit the spectral shape space to characterize both inter‐patient anatomical variability and intra‐patient changes across treatment sessions, which enables qualitative comparisons and visualizations of breast deformation.
  – Extrinsic (quantitative) variability: Using the intra‐patient correspondence, we rigidly align all scan surfaces to their respective CT skin surface, and then computed localized breast shape changes across the surface as well as the associated volume variations. This enables a detailed spatial and volumetric characterization of deformation patterns throughout the radiotherapy sessions.

The proposed intra‐patient correspondences pipeline is described in Section 2.3.1. We first down‐sample and clean each surface by removing noise, smoothing, and ensuring that it consists of a single connected mesh component. Then, we initialize pairwise correspondences between the CT skin surface and each optical surface scan of a same patient, followed by *scan‐to‐scan* correspondences between consecutive surface scans. The vertex‐to‐point correspondence computation is performed by refining the Nearest Neighbors correspondences using the ZoomOut approach.^13^ We then apply the shape collection refinement method based on cycle‐consistency from P. Galmiche et al.^14^ to compute the final intra‐patient nonrigid correspondences, with a focus on the annotated breast region as the Region on Interest (ROI).

With the ensemble of intra‐patient correspondences, we next compute inter‐patient correspondences, mapping all surfaces in the dataset to a unified space (Section 2.3.2). This allows us to identify common shape changes shared among all patients during radiotherapy, as described in Section 2.4.

### 2.3 Correspondence computation

#### 2.3.1 Intra‐patient correspondences

First, the surface data acquired by a hand‐held scanner contain considerable noise. This makes feature‐based matching methods unsuitable, as they rely on local descriptors that often vary inconsistently across corresponding anatomical regions. Second, each surface scan is a partial measurement of the participant's torso captured with a hand‐held scanner, and exhibits varying coverage. Computing correspondences between surfaces with inconsistent coverage is particularly complex, as it involves a much larger solution space compared to cases where shapes represent complete objects. To address this, we rely on a groupwise shape correspondence refinement focused on the breast region as the region of interest (ROI).^14^

**FIGURE 3:**
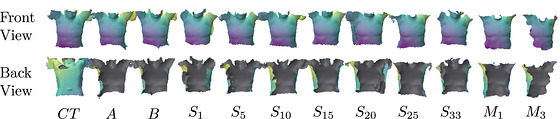
An example of the CT‐to‐scan initial correspondences, represented by color maps. Note that all scan surfaces cover only the front part of the torso, except for the CT skin surface which also includes the backside.

**Initial correspondence**. To obtain initial pairwise correspondences, we begin by manually aligning the scan acquisitions to the CT skin surface. Methods like partial shape matching techniques^15^ yielded unsatisfactory results for shape pairs containing considerable noise and coverage differences. From this alignment, initial point‐wise correspondences were defined based on Euclidean proximity—each point on a scan $S_{i}$ was matched to its closest point on the CT skin surface. This procedure provided initial correspondences of sufficiently high quality, despite variations in coverage between scans. Figure 3 illustrates the result of these initial scan‐to‐CT correspondences, where each point on the scan surface inherits the color of its corresponding point on the reference CT skin surface. The resulting correspondences are smooth and anatomically consistent, even in challenging cases such as between the CT and $M_{3}$, where only the left side of the torso was acquired.

**Group‐wise correspondence refinement**. These initial correspondences are subject to alignment uncertainties and errors induced by nonrigid deformations of the patient torso. To refine them by considering all the shapes of a given patient, we employ a spectral approach similar to that of P. Galmiche et al.^14^ Based on the functional map framework, this method iteratively refines functional correspondences between the patient shapes until they are cycle‐consistent.^16^, ^17^, ^18^, ^19^ That is, the composition of maps forming cycles (i.e., starting and ending on the same shape) approximates the identity. Additionally, it enforces map consistency within a region of interest (ROI), which, in our case, is the treated breast. As detailed in Appendix A.2, the ROI is initially defined by projecting the PTV (Planning Target Volume) onto the CT skin surface. The appendix also describes our method for cycle‐consistent correspondence refinement using functional maps.

#### 2.3.2 Inter‐patient correspondences

**FIGURE 4:**
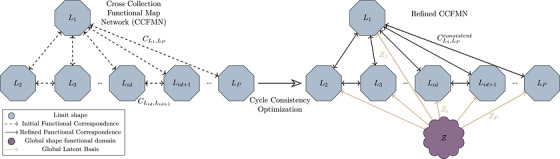
Cross collection Function Map Network (CCFMN) graph, before (left) and after (right) cycle‐consistency optimization. The output after the functional maps consistency optimization is a latent space Z representing the functional domain of a global shape and refined functional maps between the average shape of each collection.

Once correspondences have been estimated across all shapes for each patient, we compute correspondences between the shapes of different patients by computing maps between the limit shapes ${\left\{L_{id}\right\}}_{id=1}^{P}$, each representing the entire collection of a patient. Intuitively, the limit shape $L_{p}$ is the latent representation of the average shape of all surfaces acquisitions (i.e., CT, A, B, $S_{1},\text{…},S_{33}$, $M_{1}$, and $M_{3}$) from patient p. To match two limit shapes of two patients, we first select the CT skin surface as a representative reference shape from each patient's collection. We then match their limit shapes using an existing spectral matching technique. By repeating this process between multiple pairs of patient collections, we build inter‐patient shape correspondences, represented as functional maps between their respective limit shapes. We propose to represent all the patients in a *Cross Collection Functional Map Network* (CCFMN), where the nodes of the graph are the limit shapes ${\left\{L_{id}\right\}}_{id=1}^{P}$ obtained from the intra‐patient correspondences refinement, and the edges are the functional maps between the limit shapes (Figure 4 (left)). From there, we construct a *global shape* Z, which represents all the limit shapes in a manner similar to how we defined the limit shape in the intra‐patient setting (Figure 4 (right)). Intuitively, the global shape Z is the latent representation of average surface shape across the entire collection, encompassing all patient data. We optimize the global latent basis (GLB) $Z={\left\{Z_{id}\right\}}_{id=1}^{P}$, representing the functional maps from the global shape to each patient's limit shape $L_{id}$, in a manner similar to that used in CCLB, by solving the optimization problem:

(1) $$\min_{Z}\sum_{i,j}∥C_{L_{i},L_{j}}Z_{i}-Z_{j}∥\text{, s.t.}\;\sum_{i}Z_{i}Z_{i}^{T}=I.$$

Our approach shares the advantage of the CCLB while avoiding the computation of maps between each shape of each collection, which would be computationally expensive. Based on the pointwise conversion method,^20^ we propose a new pointwise map conversion procedure that utilizes both the CCLB ${\left\{Y_{i}\right\}}_{i=1}^{n}$ and the GLB ${\left\{Z_{id}\right\}}_{id=1}^{P}$ to embed all patient shapes into the global shape spectral domain Z. For more details can be found in Appendix A.2.

### 2.4 Analysis of shape changes

To study shape changes during radiotherapy, we perform both intrinsic and extrinsic analyses of the collected shapes. These analyses leverage the intra‐ and inter‐patient correspondences described previously and provide complementary insights into how the breast evolves over time. The intrinsic analysis (Section 2.4.1) evaluates deformation patterns directly on the shape manifolds. This analysis is invariant to rigid transformations and does not require alignment in Euclidean space. It enables the identification of regions with high conformal (i.e., bending) or area distortions (i.e., stretching), such as the treated breast or adjacent anatomical structures. In contrast, the extrinsic analysis estimates pointwise surface displacements and volume changes by aligning each session's scan to the planning CT skin surface. This approach provides quantitative measurements such as displacement amplitudes (in mm) and relative volume changes (in %) over time. We present these approaches in the following sections.

#### 2.4.1 Intrinsic variability analysis

To evaluate shape changes across radiotherapy sessions, we perform an intrinsic shape analysis using functional map representations derived from each patient's surface data. This approach allows us to study geometric variability without requiring explicit alignment in Euclidean space. We conduct an intra‐ and inter‐patient intrinsic shape analysis using the limit and global shape functional representations, respectively constructed in Sections 2.3.1 and 2.3.2. Specifically, we identify area and conformal deformations on a given shape $S_{n}$ by employing Characteristic Shape Differences (CSDs).^21^ See Appendix A.3 for more details.

Our framework allows to customize the shape variability analysis depending on what the user wants to study. Based on this framework, we developed an intra‐patient variability measure that allows to identify regions of shape change across all acquisitions of a same patient (Equation (A.3)), as well as an inter‐session variability measure to highlight differences among specific groups of shapes, where each group consists of acquisitions from different patients taken during a same session. The formulation for the latter analysis is given in Equation (A6) in Appendix.

#### 2.4.2 Extrinsic variability analysis on ROI

While intrinsic analysis effectively studies shape variations without alignment using scalable functional maps, it does not provide absolute measurements in the Euclidean space where deformations occur. This motivates our complementary extrinsic analysis, in which we quantify surface displacements and volume changes in the Euclidean space, relative to the planning session. For each follow‐up scan of a patient, the breast surface is rigidly aligned to the planning CT surface correspondences across the ROIs, which is obtained from the projected Planning Target Volume (PTV). This alignment enables the computation of pointwise displacements between the surfaces across sessions. From these aligned surfaces, we derive:

– Per‐vertex displacement vectors over the ROI;

– Displacement magnitudes, summarized using statistical indicators (mean, max);

– Volume change estimates, computed over the ROI region.

**FIGURE 5:**
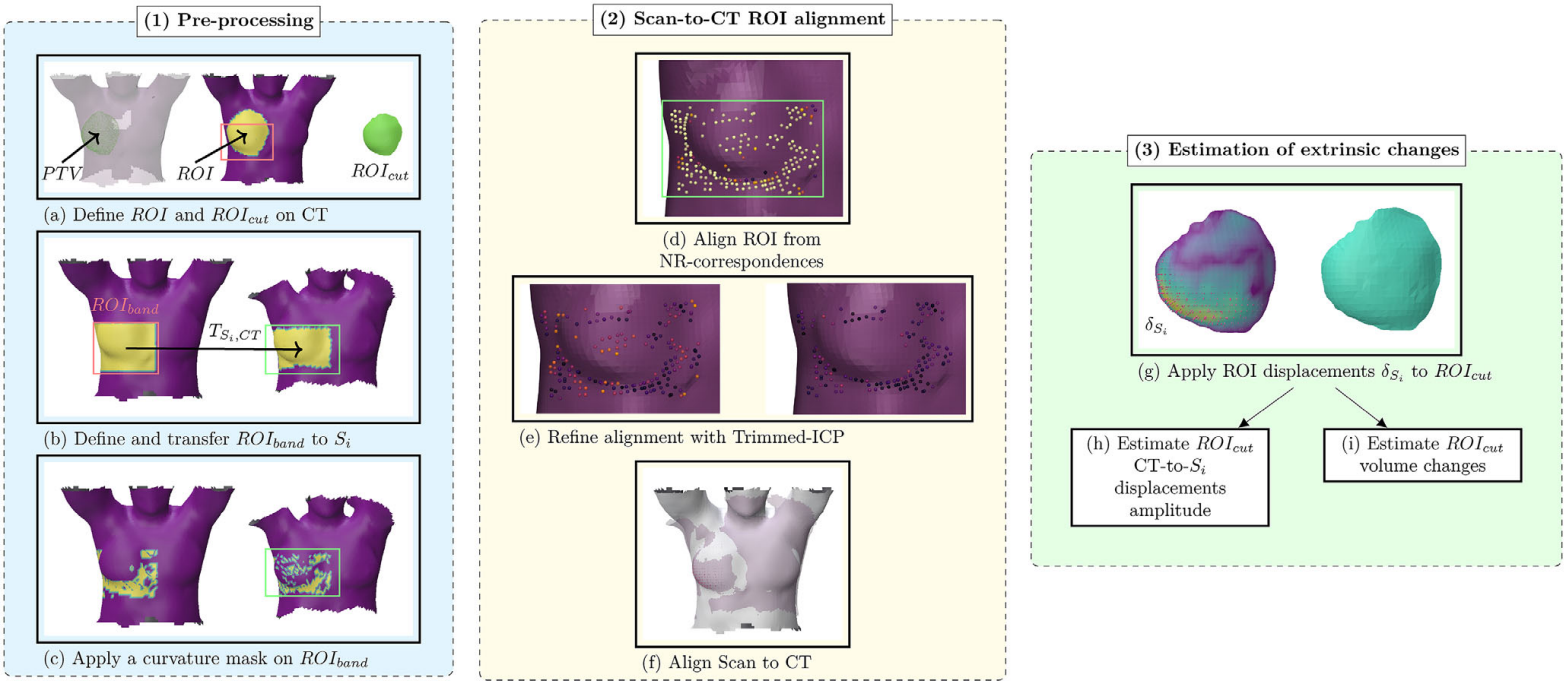
Estimation of extrinsic changes. (a) The treated breast region (ROI) is extracted from CT data, which is closed to form $ROI_{cut}$. To facilitate rigid alignment, the ROI is expanded into an $ROI_{band}$. (b) The scan‐to‐CT alignment process involves initializing with nonrigid correspondences, followed by refinement using a trimmed‐ICP on the selected points. (c) Finally, we measure the point‐to‐surface displacement vectors $\delta_{S_{i}}$ from the CT scans and estimate the resulting volume changes on the $ROI_{cut}$.

Figure 5 illustrates the workflow for estimating extrinsic surface changes in the ROI. Details of the alignment strategy, displacement field computation, and volume estimation are provided in Appendix A.4.

## 3 RESULTS

We present our results in two parts. First, we conduct an intrinsic shape analysis to qualitatively highlight changes in the torso surface during treatment. Next, we utilize nonrigid correspondences to compute displacement vectors from the CT skin to the multiple session‐wise shapes, which are then used to quantify breast shape changes.

### 3.1 Intrinsic variability analysis results

We utilize the global shape functional domain and the related global CSD introduced in section 2.4.1 to explore the shape variability at two levels: intra‐patient, and inter‐patient.

#### 3.1.1 Inter‐session analysis

**FIGURE 6:**
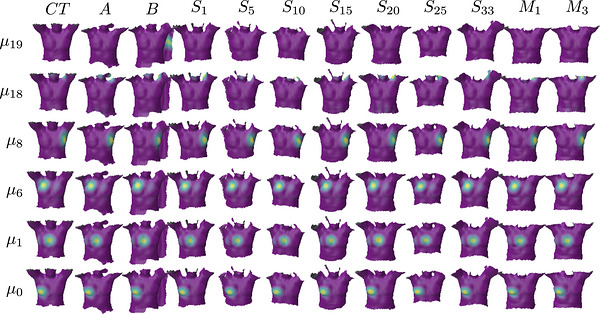
Distinctive regions $\mu_{i}$ of patient LB06. Each line highlights a region $\mu_{i}$ where there is a global area variability when all sessions are considered.

Figure 6 highlights regions of high variability among a patient collection, associated with each eigenfunctions of Equation (A7). We observe that the most distinctive regions associated with $\mu_{19}$ and $\mu_{18}$ primarily highlight the table and the shoulder regions that are not common to all shapes. In contrast, lower‐order modes provide more clinically relevant information within the ROI — $\mu_{8}$ captures area changes in the treated breast, $\mu_{6}$ and $\mu_{0}$ changes in the contralateral breast, and $\mu_{1}$ deformations on the sternum. It confirms that the region of highest variability is not necessarily the most informative region to highlight when dealing with noisy data. Based on this observation, we analyzed the distinctive functions $\mu_{i}$ and retained only those that did not highlight noisy regions – such as the table artifact in Figure 6, where $\mu_{19}$ captures variability related to the table in acquisition B. In practice, we truncated the distinctive functions (Equation (A7)) at the first index containing noise, eliminating the noise variability from our shape analysis.

#### 3.1.2 Inter‐patient analysis

**FIGURE 7:**
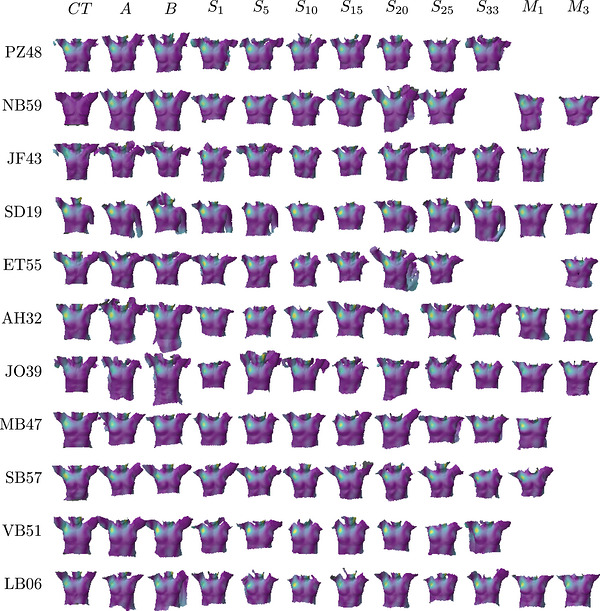
Inter‐session area variability on patient treated for right‐breast radiotherapy. The distinctive function, truncated at the 7th index is shown on all the shapes, with higher values in yellow.

**FIGURE 8:**
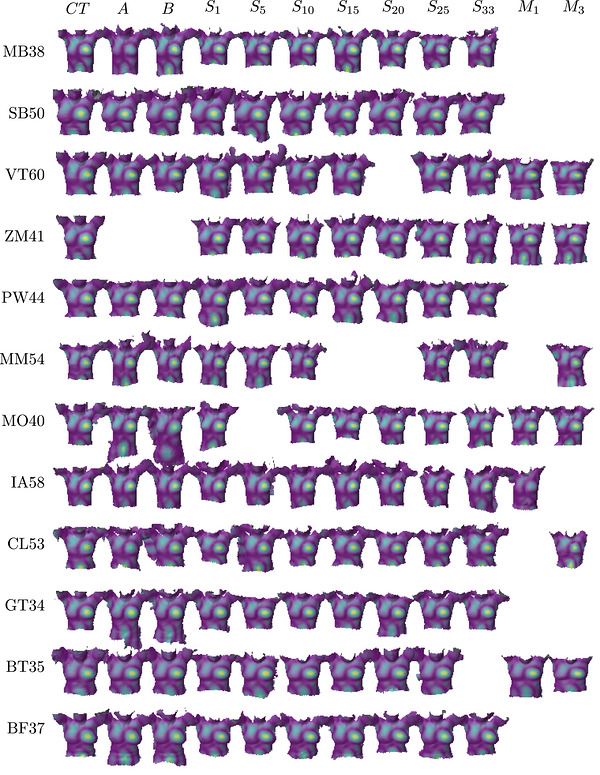
Inter‐session conformal variability in patients undergoing treatment for the left breast. The inter‐session conformal distinctive function, truncated at the 13th index, is shown on all the shapes with highly distinctive regions in yellow.

We performed two types of inter‐patient analysis. First, we considered all the acquisitions of several patients as one shape collection and measured global variability. Next, we designed an inter‐session shape variability analysis, aiming at identifying breast change patterns occurring between sessions while minimizing the contribution of inter‐patient shape differences. We employed the distinctive functions defined in Equation (A7) to localize the regions contributing the most to shape variability and display them in Figures 7 and 8. They illustrate an inter‐collection shape analysis, where each collection groups all patient acquisitions from a treatment session. This organization yields 12 session‐based shape collections. Using Equation (A6), we specifically measured inter‐session variability while minimizing inter‐patient differences within each session. More details are provided in Appendix A.3. These results demonstrate that the treated breast region showed the most prominent distortions throughout the treatment.

#### 3.1.3 Validation on synthetic data

To validate our method, we have conducted experiments using synthetic data, as in Galmiche and Seo.^14^ Validation on a physical phantom was challenging, particularly because achieving controlled deformation is nearly impossible, making it difficult to obtain reliable ground truth data. Moreover, it is difficult to separate the measurement error from the computational error. We circumvent this limitation by using synthetic mesh data from a public dataset, for which the ground‐truth correspondences are known. The correspondences are transferred in a cyclic fashion across a set of shapes, from $S_{0}$ to $S_{1}$,..., and back to $S_{0}$. The error is then computed as the average geodesic distance between each vertex and its corresponding counterpart. Our method resulted in an average error of 0.39 mm when tested on five synthetic datasets of woman's torso.

### 3.2 Extrinsic analysis results

**FIGURE 9:**
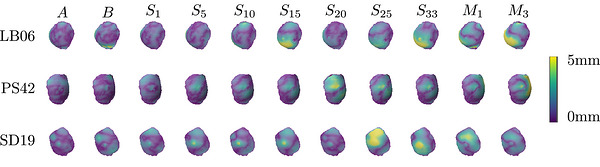
$ROI_{cut}$ has been deformed to match the $ROI_{S_{i}}$. The colormap visualizes the amplitude of the displacement vectors.

Figure 9 presents the extracted breast region, denoted as $ROI_{cut}$, deformed to match the various sessions on three different patients. Yellow regions indicate areas of higher displacement amplitudes (5*mm*). The deformations vary depending on the session and patient; for example, LB06 (first row) exhibits localized deformations at the bottom of the breast in sessions $S_{15}$, $S_{25}$, and $S_{33}$, while PS42 (second row) shows more widespread variations across the entire breast. For patient SD19 (third row), the presence of nipples is depicted as a yellow point in the middle of the deformed breasts, a result of the absence of the nipple in the pre‐processed centering CT skin surface. This qualitative analysis effectively highlights the regions where the treated breast experiences the most deformation, visually showing an increase in breast displacements for SD19 during session $S_{25}$.

To quantify the deformations in the breast region defined in Section 2.3.1, we calculated the average displacement vector amplitudes, as shown in Table 1. The average and standard deviation of displacement lengths compared to the ROI of the initial CT image are presented for each (patient, session) pair in millimeters. Notably, session $S_{25}$ for patient SD19 exhibits the highest deformation, with average displacements around 3 mm in the breast region, compared to approximately 1 mm in other sessions. This quantitative analysis confirms the increased deformations observed qualitatively for SD19 during this session. Overall, we find that the treated breast undergoes nonrigid surface displacements of approximately 2 mm on average, with increased variability observed at the end of the treatment (session $S_{25}$) and in the months following the treatment (M1 and M3).

**TABLE 1:** ROI displacements amplitude in millimeters.

	CT ROI vol (mL)	SA	SB	S1	S5	S10	S15	S20	S25	S33	M1	M3	Sessions avg
SD19	301.71	0.64 ± 0.58	1.01 ± 0.77	0.95 ± 0.74	1.14 ± 0.83	1.03 ± 0.84	1.37 ± 0.92	0.70 ± 0.55	3.06 ± 1.90	**2.19 ± 1.37**	1.95 ± 1.16	1.18 ± 0.71	1.46 ± 0.73
JO39	333.40	1.48 ± 0.96	0.90 ± 0.59	1.55 ± 1.01	1.37 ± 0.99	1.99 ± 1.40	1.50 ± 0.95	1.83 ± 1.40	**3.07 ± 2.37**	1.57 ± 1.37	1.48 ± 1.01	**3.62** ± **2.74**	1.89 ± 0.83
MB52	369.17	0.83 ± 0.74	0.65 ± 0.57	1.26 ± 0.98	1.22 ± 1.20	0.81 ± 0.72	0.89 ± 0.68	0.73 ± 0.57	1.32 ± 1.34	**1.50 ± 1.21**	1.24 ± 0.85	**2.74** ± **2.55**	1.24 ± 0.60
NB59	380.83	0.91 ± 0.79	1.14 ± 1.00	1.88 ± 1.30	1.03 ± 0.77	1.12 ± 0.70	0.98 ± 0.81	1.09 ± 0.96	1.47 ± 1.12	—	**1.98 ± 1.54**	**2.00** ± **1.50**	1.41 ± 0.43
PS42	397.75	0.80 ± 0.59	0.56 ± 0.66	0.76 ± 0.50	0.81 ± 0.72	0.84 ± 0.68	0.88 ± 0.72	**2.08 ± 1.24**	1.44 ± 1.07	0.91 ± 0.78	1.47 ± 1.20	**2.26** ± **1.93**	1.20 ± 0.59
MO40	435.22	0.96 ± 0.69	**3.18** ± **1.92**	1.06 ± 0.64	—	0.65 ± 0.51	0.86 ± 0.92	1.11 ± 0.96	0.83 ± 0.86	0.81 ± 0.63	1.75 ± 1.22	**2.00 ± 1.51**	1.36 ± 0.82
MB38	482.86	0.45 ± 0.32	0.44 ± 0.35	1.74 ± 1.38	2.48 ± 2.48	2.34 ± 1.33	1.10 ± 1.00	**4.15 ± 3.18**	**5.58** ± **4.65**	3.71 ± 2.67	—	—	**2.69** ± **1.70**
LB06	489.94	0.98 ± 1.22	0.95 ± 1.01	0.69 ± 0.70	0.94 ± 1.09	1.29 ± 1.34	1.99 ± 1.40	0.93 ± 0.95	1.78 ± 1.30	1.61 ± 1.17	**2.25 ± 1.83**	**2.93** ± **2.58**	1.54 ± 0.71
PZ48	495.18	0.81 ± 0.67	**0.94 ± 0.75**	0.59 ± 0.55	0.80 ± 0.66	0.76 ± 0.62	**1.19** ± **0.94**	0.82 ± 0.71	—	0.58 ± 0.54	—	—	0.81 ± 0.21
IA58	497.86	1.22 ± 0.73	0.92 ± 0.66	**2.37 ± 1.79**	1.88 ± 1.28	1.23 ± 0.89	0.93 ± 0.73	1.54 ± 0.95	2.06 ± 1.40	1.10 ± 0.81	**2.80** ± **2.24**	—	1.65 ± 0.67
PW44	513.38	1.52 ± 0.94	0.73 ± 0.58	1.11 ± 1.15	1.52 ± 1.07	1.19 ± 1.08	0.89 ± 0.73	2.03 ± 1.70	**2.05 ± 1.74**	**2.20** ± **1.43**	—	—	1.46 ± 0.57
GT34	555.85	1.04 ± 0.75	1.03 ± 0.70	0.93 ± 0.64	0.95 ± 0.63	**1.52 ± 1.15**	1.09 ± 0.80	**1.92** ± **1.31**	1.40 ± 1.15	1.14 ± 0.94	—	—	1.25 ± 0.34
JF43	575.26	**2.11 ± 1.85**	1.48 ± 1.07	**2.77** ± **2.50**	0.96 ± 0.87	1.71 ± 1.38	1.57 ± 1.52	1.66 ± 1.82	1.68 ± 1.36	1.08 ± 0.83	1.55 ± 1.44	—	1.61 ± 0.51
ZM41	686.05	—	—	**1.09 ± 0.89**	0.86 ± 0.72	0.91 ± 0.87	0.98 ± 0.98	0.90 ± 0.67	0.78 ± 0.69	0.96 ± 0.69	**2.88** ± **2.80**	1.01 ± 0.99	1.15 ± 0.65
MM54	698.51	0.65 ± 0.58	0.98 ± 0.90	1.46 ± 1.77	1.02 ± 1.05	**2.25** ± **1.76**	—	—	**1.94 ± 1.84**	1.77 ± 1.35	—	1.76 ± 1.90	1.60 ± 0.47
SD56	700.37	1.62 ± 1.40	1.37 ± 1.21	**2.13 ± 1.92**	1.85 ± 1.23	1.24 ± 1.01	0.94 ± 0.97	1.46 ± 1.49	1.64 ± 1.44	—	—	**4.60** ± **4.48**	1.90 ± 1.15
BT35	722.53	1.10 ± 1.05	1.10 ± 1.03	1.35 ± 1.29	1.49 ± 1.53	**2.23 ± 1.99**	2.07 ± 1.95	1.93 ± 1.98	2.14 ± 2.29	—	1.42 ± 1.44	**3.38** ± **3.15**	1.90 ± 0.68
MB47	757.91	1.97 ± 1.66	1.54 ± 1.20	**2.89 ± 1.85**	1.99 ± 1.55	2.50 ± 2.17	1.69 ± 1.21	2.53 ± 2.17	1.81 ± 1.46	2.18 ± 1.42	**3.00** ± **2.06**	—	**2.24 ± 0.52**
LD49	808.94	0.60 ± 0.53	0.51 ± 0.43	1.30 ± 1.06	**1.66 ± 1.47**	0.93 ± 0.98	1.53 ± 1.04	1.28 ± 1.10	0.99 ± 0.88	—	1.35 ± 1.13	**6.82** ± **6.04**	1.82 ± 1.91
AF45	877.28	2.21 ± 2.02	**2.26 ± 1.89**	1.51 ± 1.27	1.63 ± 1.14	1.68 ± 1.50	1.33 ± 1.16	1.56 ± 1.38	1.27 ± 1.05	**2.62** ± **1.82**	—	—	1.73 ± 0.47
SD03	1185.07	1.14 ± 0.88	1.10 ± 1.12	2.23 ± 1.70	1.60 ± 0.98	2.39 ± 1.43	0.75 ± 0.70	**3.23** ± **2.99**	1.14 ± 1.10	—	**2.59 ± 1.84**	2.35 ± 1.85	1.93 ± 0.83
SB50	1210.52	**2.47 ± 1.86**	**2.52** ± **1.68**	1.59 ± 1.20	1.31 ± 0.96	1.78 ± 1.39	1.81 ± 1.24	1.79 ± 1.38	1.23 ± 1.04	1.69 ± 1.23	—	—	1.71 ± 0.39
Patients avg	612.53	1.21 ± 0.58	1.20 ± 0.69	1.51 ± 0.64	1.36 ± 0.45	1.47 ± 0.60	1.25 ± 0.40	1.68 ± 0.85	1.84 ± 1.05	1.62 ± 0.78	**1.98 ± 0.62**	**2.82** ± **1.56**	1.62 ± 0.72

*Note*: The average and standard deviation of the displacement vectors length are provided for each patient session. The three highest values are highlighted as follows: the **highest value** is bold and underlined, the **second‐highest** is bold, and the third‐highest is underlined.

Table 2 presents the relative volume changes (%) with respect to the reference surface, observed across various sessions. Notably, session $S_{25}$ for patient SD19 shows the largest change, with a potential 18% increase in breast volume. It is important to note that this patient had the smallest initial volume, indicating that a small absolute change resulted in a large relative difference.

**TABLE 2:** Treated Breast ROI relative volume change in percentage.

	CT ROI vol (mL)	SA	SB	S1	S5	S10	S15	S20	S25	S33	M1	M3	Sessions avg
SD19	301.71	0.98	0.52	4.00	5.52	2.02	6.45	1.87	**18.23**	**13.50**	−7.65	−4.68	6.44 ± 3.14
JO39	333.40	−5.29	−4.03	−0.43	−7.44	−10.54	−4.86	−9.47	**−18.20**	−7.89	−6.17	**−21.82**	**9.09** ± **2.19**
MB52	369.17	−2.54	−1.38	**−5.61**	3.70	−1.90	3.34	−0.05	4.16	0.26	0.98	**15.18**	3.66 ± 3.49
NB59	380.83	−0.53	2.07	**7.70**	2.98	3.33	0.87	1.72	4.37	—	2.50	**7.99**	3.72 ± 2.07
PS42	397.75	3.20	−1.97	3.21	−2.01	0.48	0.70	−3.56	−0.67	−0.24	**6.02**	**9.45**	2.83 ± 1.72
MO40	435.22	0.30	**6.76**	−1.95	—	0.15	−1.07	−1.14	−1.94	−0.70	−2.73	**7.10**	2.62 ± 2.07
MB38	482.86	1.58	1.16	1.52	8.56	−4.09	1.94	**−12.99**	**−22.12**	−4.72	—	—	**7.14 ± 1.50**
LB06	489.94	1.65	−0.28	0.92	0.97	0.29	**6.06**	2.18	5.29	3.79	2.37	**10.07**	3.22 ± 1.84
PZ48	495.18	−1.25	0.56	−0.27	**−2.57**	**−1.79**	−0.29	−1.06	—	−0.50	—	—	1.01 ± 6.48
IA58	497.86	2.38	1.81	**5.02**	4.50	**4.54**	0.25	3.68	0.02	3.09	−2.52	—	2.82 ± 6.90
PW44	513.38	−1.40	0.39	3.79	−1.76	−2.21	0.98	**4.08**	**6.30**	−0.45	—	—	2.50 ± 7.27
GT34	555.85	0.11	0.01	0.59	0.53	−3.45	−1.33	**−5.25**	**−4.32**	−0.13	—	—	1.95 ± 2.39
JF43	575.26	2.86	**3.76**	**6.99**	0.50	3.53	3.13	−0.04	3.62	1.81	−0.63	—	2.67 ± 4.43
ZM41	686.05	—	—	1.28	−0.70	0.70	**2.12**	0.77	0.50	1.62	**11.42**	−0.94	2.23 ± 2.56
MM54	698.51	−1.38	−0.43	1.74	**−3.01**	−1.94	—	—	**−5.79**	−1.27	—	2.79	2.42 ± 2.54
SD56	700.37	6.97	5.87	**9.28**	4.47	1.57	−0.61	1.21	3.72	—	—	**−14.81**	5.19 ± 2.94
BT35	722.53	−1.59	−1.89	−3.12	−4.22	−5.83	−6.06	−4.77	**−6.37**	—	−3.97	**−8.94**	5.02 ± 2.08
MB47	757.91	−3.32	−1.25	−4.21	1.09	**−6.52**	−1.30	−3.53	2.00	−4.37	**−7.65**	—	3.55 ± 0.87
LD49	808.94	−1.27	−0.70	2.86	**4.63**	2.37	0.49	3.52	2.37	—	2.61	**22.78**	4.70 ± 0.57
AF45	877.28	**−2.83**	**−4.97**	2.00	−0.08	1.88	0.81	−0.91	−0.81	1.13	—	—	1.57 ± 2.14
SD03	1185.07	2.63	−2.51	**5.36**	0.55	−0.14	−0.49	−4.38	−0.25	—	**5.07**	−2.59	2.37 ± 5.54
SB50	1210.52	−1.59	−0.05	−0.90	−1.00	**−1.69**	−1.26	**−1.79**	−1.15	1.64	—	—	1.18 ± 4.81
Patients avg	612.53	2.17 ± 1.62	2.02 ± 1.97	3.31 ± 2.51	2.89 ± 2.37	2.77 ± 2.45	2.11 ± 2.05	3.24 ± 3.13	**5.34 ± 6.30**	2.77 ± 3.46	4.45 ± 3.04	**9.93** ± **6.98**	3.54 ± 3.16

*Note*: The volume initial volume $V(ROI_{closed})$ is given for each patient in the first column. The other columns report the relative volume differences $V(ROI_{S_{i}})$ for each session, relatively to the volume of the CT. The three highest values are highlighted as follows: the **highest value** is bold and underlined, the **second‐highest** is bold, and the third‐highest is underlined.

## 4 DISCUSSION

### 4.1 Functional map framework for cross‐collection shape modeling

The proposed method, built upon a functional representation, has been successfully applied to a challenging clinical trial dataset consisting of several hundred shapes of women's torso with varying coverage and noise. To the best of our knowledge, this is the first trial to utilize functional maps on such a unique dataset. It is also the first computational approach to study the breast shape and volume change during the radiotherapy both quantitatively and qualitatively. We believe that the detailed understanding of shape change can contribute to an improve personalized radiotherapy treatment, such dynamic dose planning over the treatment or patient positioning.

### 4.2 Comparison with commercial SGRT

In this study, we used a handheld 3D scanner due to its portability, ease of use, and availability in the absence of a commercial SGRT system. While our method relies on the same type of data as SGRT (Surface Guided Radiation Therapy)—namely, surface geometry—it was developed within a different context. SGRT is primarily concerned with in‐therapy acquisition and monitoring, whereas our approach focuses on the longitudinal analysis of shape and volume change across multiple therapy sessions. To our knowledge, this represents the first systematic attempt to address this problem in such a manner. As such, our method is complementary to SGRT and holds potential for adaptation to in‐therapy settings. In particular, it can be used to predict or detect significant breast volume changes over the course of radiotherapy—changes that may trigger dose recalculation and can be taken into account at the time of dose calculation. This is despite that fact that the surface data used in this study contains measurement errors on the captured surface geometry.

### 4.3 Clinical implications

Although based on surface data, our results are consistent with those reported by T. Alderliesten et al.^1^ and J. Seppälä et al.,^2^ who suggested that surface data may serve as a reliable indicator or surrogate for volumetric imaging data. In this context, our work also aligns with the perspective of P. Freisledere et al.^22^ who suggested the use of deformable surface registration as a promising avenue for supporting personalized patient treatments. Further to these findings, our approach offers a useful tool for automatically monitoring extrinsic changes in patients during each session and identifying sessions that deviate from the norm.

### 4.4 Limitations

The results obtained in this study are subject to measurement errors inherent in the scan data, primarily arising from patient movement during acquisition and the operator‐dependent nature of the process. More stable acquisition setups, such as those used in SGRT systems, could mitigate these limitations by enabling substantially faster acquisition times and thereby improving the reliability of the captured surface geometry.

### 4.5 Future directions: Assessing dosimetric impact and clinical generalizability

To assess the clinical utility of the observed surface and volume changes, a prospective study would be necessary to evaluate their impact on dose distribution. This would require integrating surface data with CT‐based dose computations, as recently proposed in other studies.^11^, ^23^ Any change in surface or volume inevitably affects the dose; the key question is to determine the extent of this impact. This is a complex issue, and providing a clear and quantified answer would require a comprehensive study. Based on our clinical experience, a surface change of 2mm or a volume variation of 4% is not necessarily sufficient to justify triggering adaptations in the treatment plan. One possible extension of our work would be to integrate automatic quantification of the dosimetric impact of surface or volume changes into the proposed pipeline. Finally, although tested here in the context of breast cancer, our framework could be extended to other cancer types where surface deformation is clinically relevant, such as skin cancer. In treatment techniques like Volumetric Modulated Arc Therapy (VMAT), where precise target positioning is essential, the ability to detect even subtle surface changes can provide key clinical value.

## 5 CONCLUSION

We developed a comprehensive pipeline to explore shape variability in a pairwise, collection, and cross‐collection manner for analyzing breast shape changes during post‐operative radiotherapy. Our analysis revealed a wide spectrum of breast changes as well as common tendencies across patients over the course of treatment, both in terms of displacement amplitudes within the target region and changes in treated breast volume. Notably, the greatest changes, on average, were observed at the 25th session. While average volume remained relatively stable across patients, some individuals exhibited significant increase. These findings highlight the importance of monitoring breast changes to better understand and respond to unexpected shape variations during radiotherapy.

Investigating the dosimetric impact of these deformations would be of great interest to clinicians, offering insights into skin irradiation and its associated side effects.

## CONFLICT OF INTEREST STATEMENT

The authors declare no conflicts of interest.

## A.1 Preliminaries

**Spectral mesh representation**. We assume a collection of N 3D shapes $S^{id}={\left\{S_{n}^{id}\right\}}_{n=1}^{N}$ for each patient $P^{id}$, represented as triangular meshes. To each shape $S_{n}^{id}$, we associate a positive semi‐definite Laplacian matrix $L_{n}=A_{n}^{-1}W_{n}$ by using the standard cotangent weight scheme of Pinkall et al.,^24^ where $W_{n}$ is the cotangent weight matrix and $A_{n}$ is the diagonal lumped area matrix. The eigendecomposition of $L_{n}$ form the spectral representation of the shape. Specifically, the basis functions $\Phi^{S_{n}}={\left\{\phi_{j}^{S_{n}}\right\}}_{j\geq 1}$ composed of the eigenfunctions of $L_{n}$ form an orthonormal basis of the square integrable functions on $S_{n}$, denoted as $L^{2}\left(S_{n}\right)$, allowing to represent any function $f\in L^{2}\left(S_{n}\right)$ using a Fourier series: $f=\sum_{j\geq 1}\left\langle f,\phi_{j}^{S_{n}}\right\rangle \phi_{j}^{S_{n}}$. We use this spectral representation of the shapes for its compactness and flexibility, as showcased by the functional map framework which we describe below.

**Functional correspondences**. The functional map framework originally presented by Ovsjanikov et al.^12^ aims at finding correspondences between shapes $S_{i}$ and $S_{j}$ as a linear transformation $\tau :L^{2}\left(S_{i}\right)\to L^{2}\left(S_{j}\right)$ between their respective function spaces. As a linear operator, τ admits a matrix representation $C=(c_{kl})$ with the coefficient determined as follows. The action of τ on a function $f\in L^{2}\left(S_{i}\right)$ can be expressed as:

(A1)
$$\begin{array}{cc} \tau f & =\tau \sum_{k\geq 1}\langle f,\phi_{k}^{S_{i}}\rangle \phi_{k}^{S_{i}}=\sum_{k\geq 1}\langle f,\phi_{k}^{S_{i}}\rangle \tau \phi_{k}^{S_{i}}\\ & =\sum_{k,l\geq 1}{\langle f,\phi_{k}^{S_{i}}\rangle }_{S_{i}}\underset{c_{kl}}{\underset{︸}{{\left\langle \tau \phi_{k}^{S_{i}},\phi_{l}^{S_{j}}\right\rangle }_{S_{j}}}}\phi_{l}^{S_{j}}. \end{array}$$

Truncating the Fourier series to the first K terms allows to obtain a particularly compact representation of the functional correspondences with a K×K matrix C, where K is chosen to be small (between 20 and 100 in practice).

A general approach to optimize such a matrix assumes the availability of corresponding functions $g_{c}\approx \tau f_{c},\;c=1,\text{…},Q$ and asks the functional map C to conserve the spectral coefficients in a least square sense:

(A2)
$$\min_{C}{∥CA-B∥}_{F}^{2}+\alpha E_{reg}\left(C\right),$$

where **A** $=(\left\langle \phi_{k}^{S_{i}},f_{c}\right\rangle )$ and **B** $=(\left\langle \phi_{l}^{S_{j}},g_{c}\right\rangle )$ are K×Q matrices of Fourier coefficients of the corresponding functions, α is a scalar weight and $E_{reg}\left(C\right)$ is a regularization term. Recovering a point‐wise map from a given functional map can be expressed as the solution of the following optimization problem:

(A3)
$$T_{ij}\left(p\right)=\underset{q\in S_{j}}{arg min}{∥C_{ji}\Phi_{k}^{S_{j}}{\left(q\right)}^{T}-\Phi_{k}^{S_{i}}{\left(p\right)}^{T}∥}_{2}\;,\;\forall p\in S_{i},$$

which can be efficiently solved by searching for each row of $\Phi^{S_{i}}$ its nearest‐neighbor in the space of rows of $\Phi^{S_{j}}$, transformed by $C_{ji}$. Indeed, any point $p\in S_{j}$ can be represented by the corresponding delta function $\delta_{p}\in L^{2}\left(S_{j}\right)$, expressed in $\Phi^{S_{j}}$ as $\Phi^{S_{j}}\left(p\right)=\left[\phi_{1}^{S_{j}}\left(p\right),\phi_{2}^{S_{j}}\left(p\right),\text{…},\phi_{K}^{S_{j}}\left(p\right)\right]$. Conversely, a functional map $C_{ji}$ can be obtained from a point‐to‐point map $T_{ij}:S_{i}\to S_{j}$ using $C_{ji}={\left(\Phi^{S_{i}}\right)}^{†}\Pi_{ij}\Phi^{S_{j}}$, where $\Pi_{ij}$ is the matrix representation of $T_{ij}$ such that $\Pi_{ij}\left(index\left(p\right),index\left(q\right)\right)=1$ if $T_{ij}\left(p\right)=q$ and 0 otherwise, with $p\in S_{i}$ and $q\in S_{j}$.

## A.2 Groupwise correspondence refinement

**(1) Identification of the Region of Interest (ROI)**: Identifying the treated breast region or ROI on all acquisitions is a key step in our study. We first locate it on the CT surface, thanks to the point cloud annotation provided by clinicians. Following this, the breast region is identified on the other scans using the correspondences between the CT and those scans. A point on a scan is considered part of the ROI if its corresponding point belongs to the ROI.

**(2) Cycle‐consistency refinement**: A common strategy to refine maps in a collection manner is to use cycle consistency constraints.^16^, ^17^, ^18^, ^19^ The main idea is to ensure that the composition of maps, starting and ending on the same shape, results in the identity. In our work, we utilize the *Canonical Consistent Latent Bases* (CCLB),^21^ where an additional regularization term has been added to ensure the $\sum_{i}Y_{i}^{T}\Lambda_{i}Y_{i}$ is a diagonal matrix in order to overcome instability in the extracted latent bases.

**Inter‐patient correspondences from CCLB**. Let $S_{i}$ be a shape of the patient id. We embed the shape $S_{i}$ into the global shape embedding Z by using the operator $\zeta_{i}=\Phi_{i}Y_{i}Z_{id}$. $\zeta_{i}$ is the composition of three spectral embedding operations: $\Phi_{i}$ converts the euclidean representation of the shape $S_{i}$ into its spectral form in $L^{2}\left(S_{i}\right)$; $Y_{i}$ projects the spectral form to the limit shape $L_{id}$ of the patient $P^{id}$; and $Z_{id}$ maps the limit shape coefficients into global shape coefficients. The point‐wise map $T_{ij}$ between two shapes $S_{i}$ and $S_{j}$ is obtained by comparing the global shape coefficients of each shape and solving:

(A4)
$$T_{ij}\left(p\right)=\underset{q\in S_{j}}{arg min}{∥\zeta_{j}{\left(q\right)}^{T}-\zeta_{i}{\left(p\right)}^{T}∥}_{2},\;\forall p\in S_{i}.$$

In practice, this is done by a nearest neighbors search of each row of $\zeta_{i}=\Phi_{i}Y_{i}Z_{id_{i}}$ among the rows of $\zeta_{j}=\Phi_{j}Y_{j}Z_{id_{j}}$.

The CCLB $Y={\left\{Y_{i}\right\}}_{i=1}^{N}$ can be interpreted as a set of functional maps, mapping from the functional domain of a *limit shape* $L_{id}$, representing the entire shape collection of patient id, to the individual $S_{i}$ in the collection. Such approach has several advantages. First, there is no need to explicitly define or create a reference shape. Instead, the abstract “limit” or “average” shape $L_{id}$ is represented only in the functional domain, through the latent bases ${\left\{Y_{i}\right\}}_{i=1}^{N}$. Second, we can directly recover functional maps between different acquisitions from the CCLB by using the formula $C_{ij}^{consistent}=Y_{j}Y_{i}^{-1},\;\forall \;i,j\;\in E$. Likewise, we can compute point‐wise maps using consistent ZoomOut approach,^20^ which reduces to nearest neighbor searches of each row of $\Psi^{S_{i}}=\Phi^{S_{i}}Y_{i}$ among the rows of $\Psi^{S_{j}}=\Phi^{S_{j}}Y_{j}$. This is solved using the following formulation:

(A5)
$$T_{ij}\left(p\right)=\underset{q\in S_{j}}{arg min}{∥\Psi^{S_{j}}{\left(q\right)}^{T}-\Psi^{S_{i}}{\left(p\right)}^{T}∥}_{2},\;\forall p\in S_{i}.$$

This allows to recover efficiently consistent pointwise maps between any pair of shapes in the collection. Lastly, the construction of the latent basis has an appealing scaling property. Given a new, previously unseen shape $S_{k}$ and a functional map $C_{i,k}$ from $S_{i}$ to $S_{k}$, where $S_{i}$ is a shape from the collection used to estimate the latent shape, the latent basis can be pushed to $S_{k}$ via $Y_{k}=C_{i,k}Y_{i}$.

## A.3 Intrinsic Variability Analysis

**Characteristic Shape Differences (CSDs)**. To quantify and visualize intrinsic shape changes, we use CSDs.^21^ For a shape $S_{n}$, its CSD relative to a reference is defined as: $D_{n}^{A}=Y_{n}^{T}Y_{n}$ for area deformations and $D_{n}^{C}=\Lambda_{0}^{+}Y_{n}^{T}\Lambda_{n}Y_{n}$ for conformal deformations, where $\Lambda_{0}$ and $\Lambda_{n}$ are the eigenvalues of the limit shape and the shape $S_{n}$, respectively, and  denotes the Moore‐Penrose pseudo‐inverse.

CSDs have many appealing properties, such as their compactness and flexibility, as they rely solely on functional maps and are independent of the shapes' discretization. Another interesting property is their functoriality, which enables the computation of a shape difference $D_{nm}$ between two shapes $S_{n}$ and $S_{m}$ as $D_{nm}=Y_{n}D_{n}^{-1}D_{m}Y_{n}^{-1}$. Additionally, their algebraic nature allows them to be represented as small matrices, thus making them easy to manipulate and compose to analyze shape variability in a personalized manner.

Global variability is modeled in such a way that, after suppressing this variability, the shapes within the collection are aligned as closely as possible. It has been demonstrated^21^ that the function $\alpha^{*}$, which maximizes the differences in norms across the collection, can be determined as the eigenfunction associated with the largest eigenvalue of the matrix $E=\sum_{n,m}{\left(D_{n}-D_{m}\right)}^{2}$, where $D_{n}$ and $D_{m}$ represent characteristic shape differences (CSD). The eigenfunctions $\left\{\mu_{k}\right\}$ of E account for the functional deformations, ordered from the most to the least important.

**Inter‐patient analysis**: To compare shapes from different patient collections in the setting of inter‐patient analysis, we first represent them in the common space Z. The CSD $D_{id,n}$ of each shape $S_{id,n}$ from each patient collection $L_{id}$ (id = 1…P, n = 1…N) is transferred to the global shape functional domain Z, to obtain a global CSD $D_{id,n}^{g}$ = $Z_{id}^{-1}D_{id,n}$. The inter‐patient global shape variability is computed in the global functional domain, with the global CSD $D_{id,n}^{g}$ and the spectrum of $F=\sum_{i,j}{\left(D_{i}^{g}-D_{j}^{g}\right)}^{2}$, similarly to the global shape variability.

**Inter‐session analysis**: In addition, we derive the global cross‐session variability by maximizing the total shape variability between all shape pairs {($S_{i,n}$, $S_{j,n}$)} from different patient collections (i≠j) but belonging to the same session collection n, and minimizing the shape variability among all shape pairs $\left\{\left(S_{id,n},S_{id,m}\right)\right\}$, (n≠m) within the same patient collection id. It is implemented by computing the eigenvalues and eigenvectors of:

(A6)
$$H=\sum_{i,j}\sum_{n}{\left(D_{i,n}^{g}-D_{j,n}^{g}\right)}^{2}-\sum_{id}\sum_{n,m}{\left(D_{id,n}^{g}-D_{id,m}^{g}\right)}^{2}.$$

This ensures a robust distinction between inter‐session and inter‐patient variability, highlighting the regions with the most change across different sessions, while minimizing inter‐patient differences.

**Intrinsic variability visualization**. To obtain a compact visualization that summarizes the primary changes over the surface, we define a distinctive function in the global shape space, similar to the method outlined in R. Huang et al.^21^ This function is expressed as:

(A7)
$$D^{global}=\sum_{k=1}^{m}\lambda_{k}^{H}\mu_{k}^{H},$$

where $\lambda_{k}^{H}$ and $\mu_{k}^{H}$ denote the eigenvalues and eigenvectors of H, and m is the truncation parameter, allowing to show intrinsic variability at various levels. By changing m to a lower value, we can remove from the display the highest variability, often observed on regions of different torso coverage or noise.

## A.4 Extrinsic variability analysis

**(1) Pre‐processing**. We begin by identifying the ROI on the CT skin surface. The planning target volume (PTV) is projected onto the CT skin surface based on the Euclidean nearest neighbor. Each ROI point on the PTV is paired with its closest point on the CT skin surface. The projected ROI region is then segmented and closed to form a closed surface, $ROI_{cut}$, representing the treated breast volume (Figure 5(i)). Next, to maximize the use of the pose‐ and respiration‐invariant part of the surface in guiding the alignment process, we define a region called $ROI_{band}$ on the CT skin surface and transfer it to each scan $S_{i}$ (Figure 5(ii)). $ROI_{band}$ is defined by the portion of the surface located within a bounding box determined by the extreme points of the ROI. $ROI_{band}$ includes regions such as the rib cage and sternum, which are beneficial for rigid alignment. Finally, we compute $ROI_{mask}=ROI_{band}-ROI$ to retain only the points outside the breast region, which is likely to undergo nonrigid deformations across sessions (Figure 5(iii)).

**(2) Rigid alignment from nonrigid correspondences**. We then align the CT skin surface with each scan surface $S_{i}$ using a goal function that minimizes the sum of distances between the two $ROI_{mask}s$ (Figure 5(iv)). The rigid alignment thus obtained is further refined using a point‐to‐surface Trimmed Iterative Closest Point (ICP) algorithm (Figure 5(v)). This step improves the alignment by iteratively excluding the farthest scan points relative to the CT, effectively removing points that could contribute to nonrigid deformations.

**(3) Estimation of breast changes**. For each optical scan $S_{i}$, we define a vector field $\delta_{S_{i}}$ on the CT skin breast surface by connecting each vertex p on the CT skin to its closest point q on the $S_{i}$. Such vertex‐to‐surface approach allows the displacements to be robust to the discretization of the aligned scan. We then estimate the volume changes by deforming the closed surface $ROI_{cut}$ onto $ROI_{S_{i}}=ROI_{cut}+\delta_{S_{i}}$, where $\delta_{S_{i}}$ is the vector field calculated for each clinical trial session $S_{i}$. From this field, we compute the relative volume differences $\Delta V^{rel}\left(S_{i}\right)$ between session $S_{i}$ and the initial volume of $ROI_{cut}$, that is, $\Delta V^{rel}\left(S_{i}\right)=\frac{V\left(ROI_{S_{i}}\right)-V\left(ROI_{cut}\right)}{V(ROI_{cut})}$.

## A.5 Implementation

**Shapes pre‐processing**. We first remesh the surface scans to approximately 10K vertices using the Meshlab^25^ Visual Computing Group surface reconstruction filter. In addition, we make sure that each measured surface is composed of only one connected component. We also extract from the planning CT scan the point cloud representing the patient's torso surface, which is then remeshed to obtain a 10K‐vertex mesh, referred to as the *CT skin surface*.

**Shape matching parameters**. We calculated initial pairwise functional maps with a size of k=65, used limit shapes with a size of $k_{l}=52$, and employed a global shape size of m=20. These parameters were selected based on preliminary tests to ensure that the spectral embeddings were large enough to effectively represent the shapes, while also avoiding excessive computational costs and potential inaccuracies caused by high‐frequency noise.

**Shape alignments**. For the rigid alignment, we utilize the Procrustes and Iterative Closest Point (ICP) algorithms provided by the *trimesh* Python library. We ran 100 iterations of ICP, applying trimming every 5 iterations to remove the two percent of points with the highest point‐to‐surface distances.
